# Urinary Luteinizing Hormone Tests: Which Concentration Threshold Best Predicts Ovulation?

**DOI:** 10.3389/fpubh.2017.00320

**Published:** 2017-11-28

**Authors:** Rene Antonio Leiva, Thomas Paul Bouchard, Saman Hasan Abdullah, René Ecochard

**Affiliations:** ^1^Bruyère Research Institute and C. T. Lamont Primary Health Care Research Centre, Department of Family Medicine, University of Ottawa, Ottawa, ON, Canada; ^2^Department of Family Medicine, University of Calgary, Calgary, AB, Canada; ^3^Service de Biostatistique, Hospices Civils de Lyon, Lyon, France; ^4^Université de Lyon, Lyon, France; ^5^Université Lyon 1, Lyon, France; ^6^Équipe Biostatistique-Santé, Laboratoire de Biométrie et Biologie Évolutive, Centre National de la Recherche Scientifique, Unité Mixte de Recherche 5558, Villeurbanne, France

**Keywords:** ovulation predictor kits, luteinizing hormone, natural family planning, fertility awareness methods, infertility, urine, ovulation, fertile window

## Abstract

**Objective:**

To study the best possible luteinizing hormone (LH) threshold to predict ovulation within the 24, 48, and 72 h.

**Design:**

Observational study.

**Setting:**

Multicenter collaborative study.

**Patients:**

A total of 107 women.

**Interventions:**

Women collected daily first morning urine for hormonal assessment and underwent serial ovarian ultrasound. This is a secondary analysis of 283 cycles.

**Main outcome measures:**

The sensitivity, specificity, positive and negative predictive values, and positive and negative likelihood ratios were estimated for varying ranges of LH thresholds. Receiver operating characteristic curves and cost–benefit ratios were used to estimate the best thresholds to predict ovulation.

**Results:**

The best scenario to predict ovulation at random was within 24 h after the first single positive test. The false-positive rate was found to increase as (1) the cycle progressed or (2) two or three consecutive tests were used, or (3) ovulation was predicted within 48 or 72 h. Testing earlier in the cycle increases the predictive value of the test. The ideal thresholds to predict ovulation ranged between 25 and 30 mIU/ml with a PPV (50–60%), NPV (98%), LR+ (20–30), and LR− (0.5). At least, one day with LH ≥25 mIU/ml followed by three negatives (LH <25) occurred before ovulation in 31% of all cycles. When used throughout the cycle and evaluated together, peak-fertility type mucus with a positive LH test ≥25 mIU/ml provides a higher specificity than either mucus or LH testing alone (97–99 vs. 77–95 vs. 91%, respectively).

**Conclusion:**

We identified that beginning LH testing earlier in the cycle (day 7) with a threshold of 25–30 mIU/ml may present the best predictive value for ovulation within 24 h. However, prediction by LH testing alone may be affected negatively by several confounding factors so LH testing alone should not be used to define the end of the fertile window. Complementary markers should be further investigated to predict ovulation and identify the fertile window. The use of the peak cervical mucus along with an LH test may provide a higher specificity and predictive value than either of them alone. We recommend that manufacturers disclose their tests’ threshold to the public.

## Introduction

Commercially affordable urinary ovulation predictor tests have become commonly used by those women wanting to become pregnant since they were first introduced in the 1980s (1). In addition, they could also be used as an adjunct to Fertility Awareness Methods (2). These rapid one-step home urinary tests attempt to predict when ovulation is about to occur by measuring the luteinizing hormone (LH) surge (3). The rise in LH in the urine is known to occur near the time when ovulation takes place during the menstrual cycle (4–6) and may not be strictly one or two days before ovulation as it was first supposed. However, as also pointed out by these studies, the LH peak is rather best described as a wave than as a peak with its surge occurring prior to ovulation; yet, LH levels may remain high after ovulation during the luteinization process. All of these factors may affect how these tests are interpreted by the user in relation to the day of ovulation.

Some studies have evaluated the validity of these tests (7–9); however, there is no published evidence indicating which urinary LH concentration level may be ideal to correlate with ovulation. As a result, there is no consensus among the different manufacturers on which threshold to use. There is a wide variation among the LH test thresholds ranging from 20 to 50 mIU/ml. Thus, in the present study, we have characterized LH thresholds to determine the ideal concentration to identify ovulation within 24, 48, and 72 h.

## Materials and Methods

### Patients

Patients were recruited from 1996 to 1997 from eight natural family planning clinics in France, Italy, Germany, Belgium, and Spain as previously reported (10). A database of information was created but due to legal–commercial disclosure agreements with the funding company (Quidel Corporation), the results could not be published until now. The inclusion criteria consisted of women aged 19–45 years with previous menstrual cycle lengths of 24–34 days. Exclusion criteria consisted of women with a consistent history of anovulatory cycles, infertility, or active hormonal treatment of infertility in the past 3 months, use of hormonal contraception or hormonal replacement in the past 3 months, abnormal cycles (polycystic ovarian syndrome or a known luteal defect), hysterectomy, tubal ligation(s), or pelvic inflammatory disease. In addition, runners and breastfeeding or postpartum mothers (<3 months) were excluded given their likelihood of anovulation.

A total of 107 women were finally included, contributing an average of three cycles. The study examined 326 cycles that have been analyzed in other studies (11). Data collected from patients included information on age, age at menarche, parity, past oral contraceptive use, lifestyle habits, such as smoking, diet, and physical activity (hours/week), sleep duration (hours/day), and stress levels (subjective assessment). Height and weight were measured and body mass index (BMI) calculated. The study was approved by the local Ethics Committee (Comite Consultatif de Protection des Personnes dans la Recherche Biomedicale de Lyon). Each of the participants gave their written informed consent, and the study procedures were carried out in accordance with the Ethical Standards for Human Experimentation established by the Declaration of Helsinki.

### Hormone Assessments

Assays were carried out on the first morning urine with two 10–12 mL aliquots frozen on the day of collection at −20°C in tubes containing gentamicin sulfate. On the day of analysis, the aliquots were thawed in a single laboratory and tested in duplicates for quantitative detection of estrone-3-glucuronide (E1G, ng/mL), pregnanediol-3a-glucuronide (PDG, ng/mL), follicle-stimulating hormone (FSH, mIU/mL), and LH (mIU/mL) using time-resolved fluorometric immunosorbent assays (Delfia). Each hormonal sample was repeated twice: the relative difference (i.e., CV) was, respectively, 5.96, 10.79, 8.66, and 7.17% for PDG, FSH, LH, and E1G. We cannot provide detailed information on assay performance except the intra-assay CV’s. These data remain within the property of the funding company.

### Ultrasound Investigations

Serial transvaginal ovarian ultrasounds with follicle measurement were performed by a single physician per center. Ovarian scanning started on the first day women observed cervical mucus or when an LH surge was detected by LH home tests (Quidel Corp.), whichever came first. Scanning was performed every other day until a follicle reached 16 mm and then daily until evidence of ovulation (the ultrasound day of ovulation, US-DO). Details regarding ultrasound investigations were previously published (12).

### Measured Outcomes

A positive LH test was defined as a test result above a defined concentration threshold. A negative LH test was defined as a test result below that threshold. Nine scenarios were analyzed in the following way: (1) whether ovulation would occur within 24, 48, or 72 h following a single positive test; (2) whether ovulation would occur with 24, 48, or 72 h following 2 days of consecutive positive tests; (3) whether ovulation would occur with 24, 48, or 72 h following 3 days of positive tests. We analyzed only ovulatory cycles as the purpose was to assess the best threshold for LH to predict ovulation.

Ovulation occurring 24, 48, or 72 h after a “positive test” was classified as true positive. Similarly, if no ovulation occurred 24, 48, or 72 h after one negative test was classified as true negative. The sensitivity (Se) was estimated as the proportion of ovulations within 24, 48, or 72 h that have a “positive test.” The specificity (Sp) was estimated as the proportion of absence of ovulation within 24, 48, or 72 h that have a “negative test.” Positive predictive value (PPV) was the proportion of ovulation within 24, 48, or 72 h following a “positive test.” Negative Predictive value (NPV) was the proportion of absence of ovulation within 24, 48, or 72 h following a negative test. Prevalence (P) of ovulation across the menstrual cycle was defined as proportion of cycles having an ovulation on a given day.

Finally, positive likelihood ratios (LR+) were defined as the ratio between Se and 1-Sp, and negative likelihood ratios (LR−) as the ratio between 1-Se and Sp. In other words, LR+ was the ratio of the proportion of cycles with ovulations and a positive test to the proportion of cycles with no ovulations and a positive test. Similarly, LR− was the ratio of the proportion of cycles with ovulation and a negative test divided by the proportion of cycles with no ovulation and a negative test.

### LH Test Models and Statistical Analysis for Interpretation of the Results

We postulated a model of using the receiver operating characteristic (ROC) curves to describe the evolution of sensitivity and specificity according to (a) different thresholds (ranging from 5 to 50 mIU/ml), (b) time to ovulation (within 24, 48, or 72 h), (c) number of LH positive days (one day, two consecutive days or three consecutive days), and (d) across the menstrual cycle taking into account the daily prevalence of ovulation. The latter was especially relevant because Se, Sp, PPV, and NPV are affected by their temporal relationship to the day of ovulation.

Two core analyses were performed: first the use of any threshold across menstrual cycle and, second, an analysis to identify the performance of a given threshold on a specific day of the cycle. Due to practical and obvious reasons, the only thresholds chosen for the second analysis were based on the commercially available tests, namely 20, 25, 30, 35, and 40 mIU/ml. However, for the sake of completeness, even though not available in the market, 15 mIU/ml levels were also included. Sensitivity and specificity were estimated with their 95% confidence intervals (CI).

We made use of a decision analysis approach (cost–benefit ratio) to further assess all the proposed thresholds (13, 14). We calculated the number of false-positive tests needed to get one true positive which we named “positive benefit net cost”—this statistic was used to identify the effect of choosing each specific threshold (15, 20, 25, 30, 35, or 40). Ideally, an optimal choice treats the benefit of true identification of ovulation with a positive test, the same as true rejection of ovulation with a negative test, i.e., we are equally motivated by true-positive and true-negative results, but taking into account the difference in consequence of false positives and false negatives through the cost–benefit ratio. For example, a lower threshold would give more true positives, but at the cost of more false positives.

We additionally tested two hypotheses. The first was whether a given threshold can confirm the end of the fertile window, i.e.,: the luteal phase starting 24 h after ovulation, by achieving three daily consecutive negative results below such threshold after having one positive result above the threshold. The second hypothesis was whether the addition of peak-type cervical mucus to a positive LH test would increase its predictive value (15). We have previously defined a four-point score for types of cervical mucus: [1] dry sensation, rough, and itchy or nothing felt/nothing seen; [2] no longer dry sensation/nothing seen; [3] damp sensation, with or without appearance of thick, creamy, whitish, yellowish, or sticky mucus; [4] wet, slippery sensation with or without the appearance of clear, stretchy mucus (similar to a raw egg white). Score 4 type mucus was defined as peak-fertility type mucus. This mucus is related to estrogen and we found that this type of mucus identified the ovulation window with 88% sensitivity (15). We calculated the Se, Sp, PPV, and NPV for a given LH threshold with the highest predictive value.

Finally, in order to verify the stability of the quality of the LH test for different populations, We carried out a multivariate regression analysis on five co-variables (Age, BMI, past use of oral contraception, sport activity, and current smoking) to investigate its impact on LH levels during the window of LH testing, i.e., from the 7th to the 20^th^ day both inclusive. This regression was a mixed linear regression to take account of (1) the daily repetition of LH measurements, (2) days being clustered within cycles, and (3) cycles being clustered within women. The dependant covariate was the natural logarithm of LH in order to bring it closer to normality. The independent covariates were age, BMI past use of oral contraception, sport activity, and current smoking, and the woman was the random effect.

All statistical analyses were performed using the library pROC of R software version 3.3.3 (The R Foundation for Statistician Computing). A *p*-value <0.05 was considered for statistical significance.

## Results

**Figure 1:**
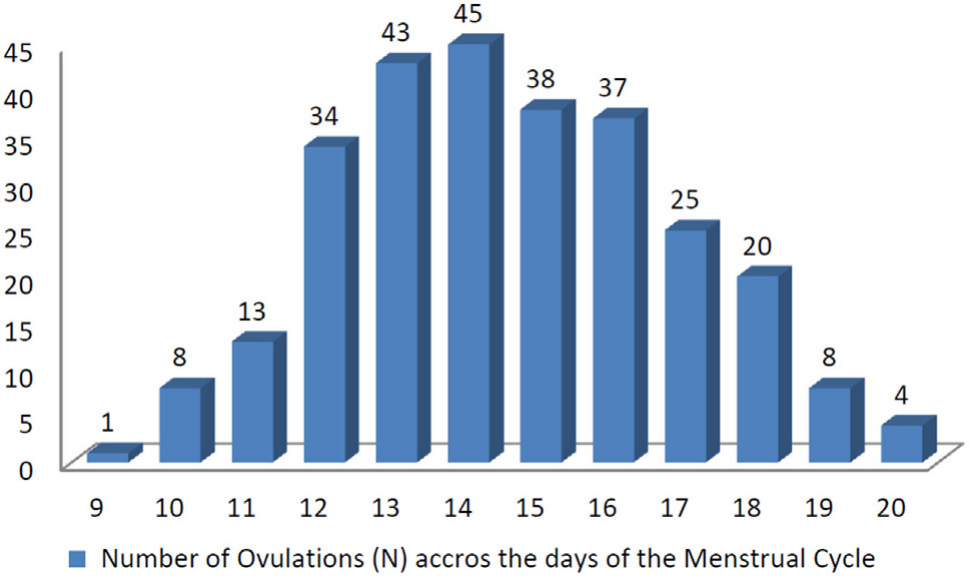
Number of ovulations (*N*) across the days of the menstrual cycle.

The characteristics of women who participated in the study and provided results for this analysis are shown in Table 1. Prevalence of ovulation is presented in Figure 1. A total of 326 cycles were used in the study. In 28 out of these 326 (9%) the first ultrasound was performed after the follicle had ruptured, so these cycles were excluded. Of the 298 remaining cycles, 15 showed no confirmation of ovulation (4.5%) which could indicate possibly Luteinized Unruptured Follicles (LUFs). We, thus, analyzed 283 cycles.

**Table 1:** Women and cycle characteristics for those with available luteinizing hormone results.

Characteristics	Mean (±SEM)	Minimum	Maximum
**Women (102)**			
Age (years)	32.43 (0.58)	19	44
Age at Menarche (years)	13.23 (0.16)	9	17
Body mass index (kg/m^2^)	21.23 (0.26)	17.12	28.34
Physical activity (hours/week)	1.13 (0.22)	0	9
Regular smokers (%)	11%		
Vegans (%)	4%		
Past use of oral contraception (%)	35%		
**Cycles (283)**			
Cycle length (days)	28.07 (0.16)	22	44
Follicular phase (days)	14.76 (0.17)	9	33
Luteal phase (days)	13.35 (0.10)	7	17

**Figure 2:**
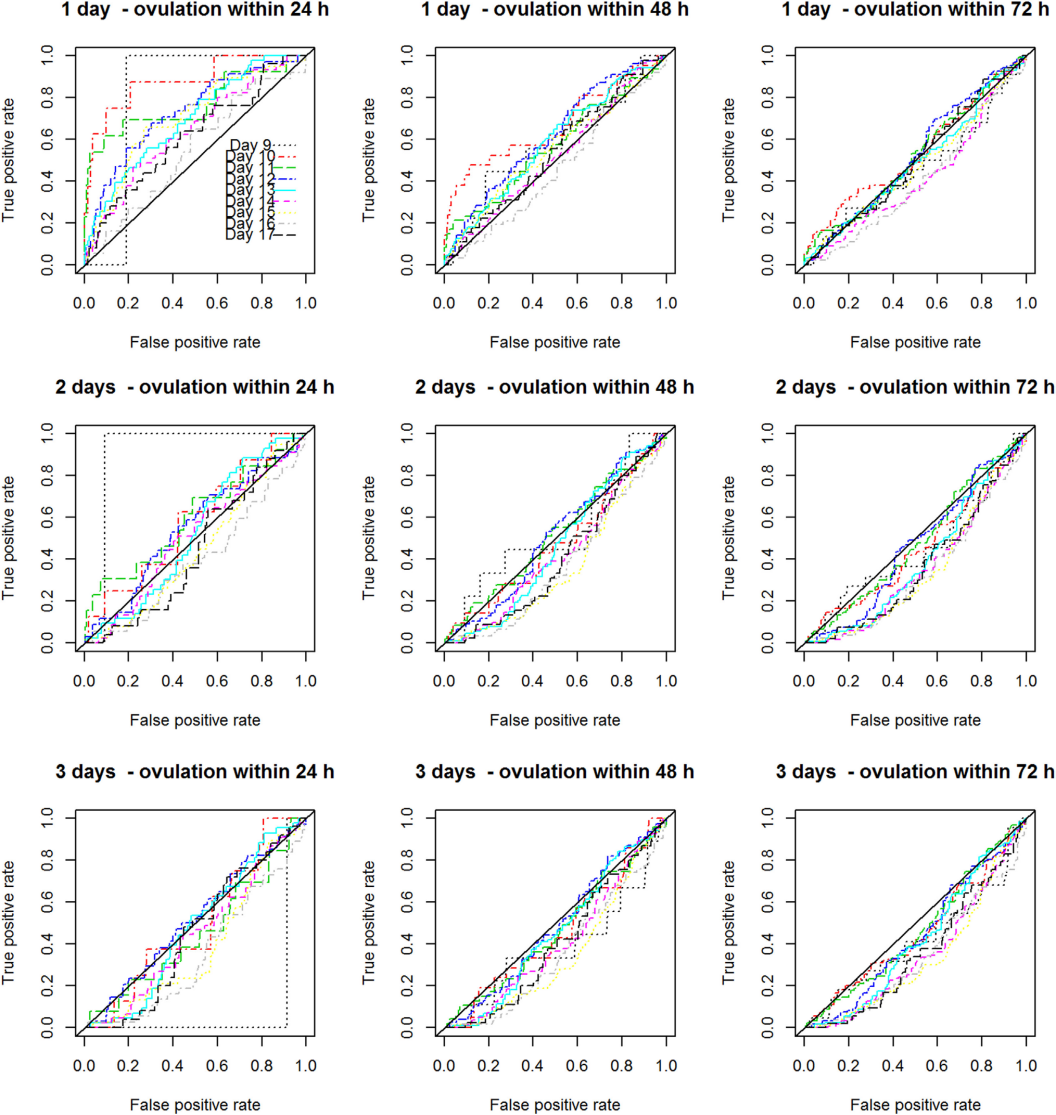
Nine ovulation predictive scenarios for the proportion of true-positive rates to false-positive rates from days 9 to 17 of the cycles.

Figure 2 displays the nine scenarios’ curves for the proportion of true-positive rates to false-positive rates from days 9 to 17 of the cycles. As one can see from these curves, the best scenario to predict ovulation at random was within 24 h after the first single positive test. The false-positive rate was found to increase as (1) the cycle progressed or (2) two or three consecutive tests were used or (3) ovulation was predicted over a longer period (48 or 72 h).

**Figure 3:**
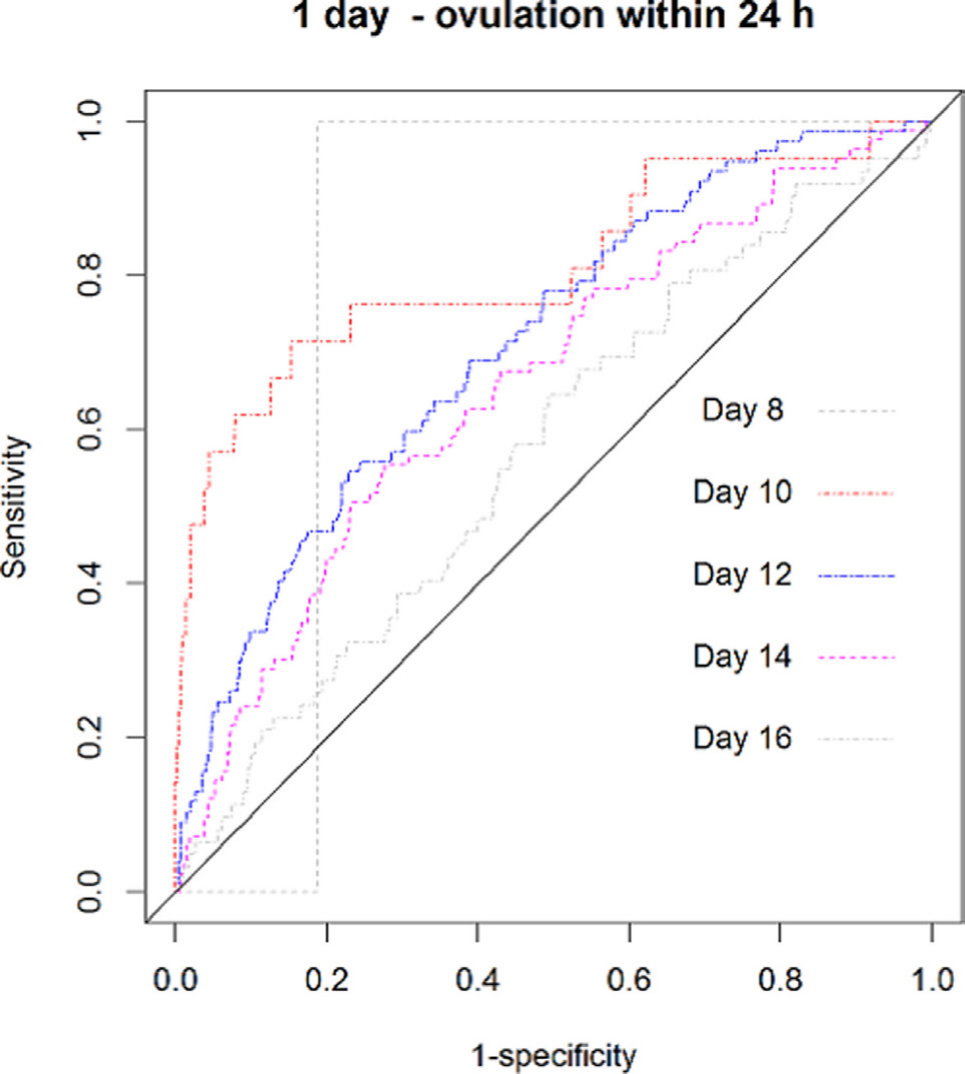
The receiver operating characteristic curves for one random positive luteinizing hormone test to predict ovulation within 24 h-across the menstrual cycle.

**Figure 4:**
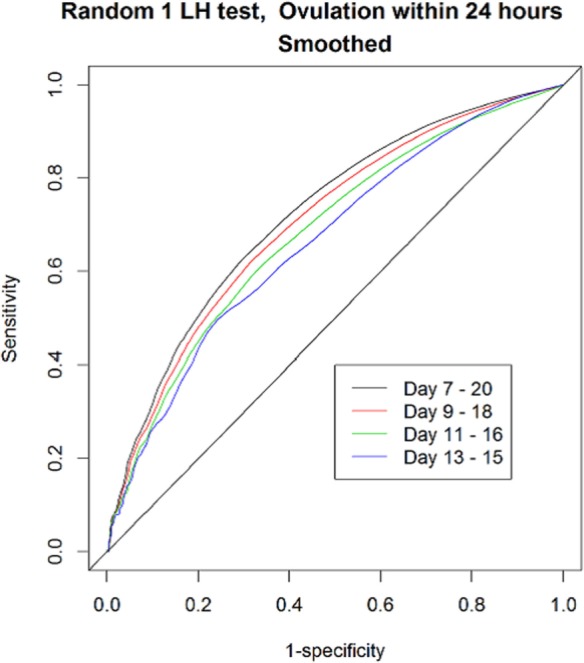
The receiver operating characteristic curves for one random positive luteinizing hormone (LH) test to predict ovulation within 24 h-across the menstrual cycle, applying different ranges on the number of daily tests used.

Figure 3 displays the ROCs for one random LH test across the cycle. In this data set, the best predictive results peak around day 10 of the cycle. In addition, the accuracy of the tests changed across the cycle, decreasing after this peak day has been achieved. If the test is used more often, e.g., daily from days 7 to 20 (13 days in total) instead of only from days 13 to 15 (3 days in total), its accuracy increases (Figure 4). Figure S6 in Supplementary Material shows all 40 graphs analyzing the Se, Sp, CIs, PPV, NPV, LH+, and LH− for the 15, 20, 25, 30, 35, and 40 mIU/ml thresholds across the menstrual cycle. This analysis further confirmed that the values found with a first single-positive test around the 10–12th day of the cycle provided the highest probability to predict ovulation within 24 h, a phase we called the “peak fertility window.” Table SA in Supplementary Material supplies the detailed statistical background for all the thresholds ranging from 0.01 to 100.

Table 2 display the “positive benefit net cost” of each threshold for predicting ovulation within the first 24 h, that is, the number of false-positives tests obtained to get one true positive. We proposed doing daily testing from day 7 until day 20. Their respective Se and Sp were also provided. These results highlighted that while a threshold of 40 mIU/ml may present the best “net cost” (one true positive while having four false positives), it does this at the expense of a very poor sensitivity (19%). It is also important to note that the thresholds 20, 25, 30, and 35 mIU/ml present similar results with a net cost of 6 and around 35% sensitivity.

**Table 2:** Positive benefit net cost for predicting ovulation within the first 24 h.

First day of test utilization	Last day of test utilization	Threshold (mIU/ml)	Positive benefit net cost	Sensitivity	Specificity
7	20	15	9	0.54	0.78
7	20	20	6	0.37	0.88
7	20	25	6	0.35	0.89
7	20	30	6	0.35	0.89
7	20	35	5	0.24	0.94
7	20	40	4	0.19	0.96

Table 3 illustrates the Se, Sp, CIs, PPV, NPV, LH+, and LH− for the 15, 20, 25, 30, 35, and 40 mIU/ml thresholds on the 11th day of the cycle, demonstrating test accuracy at peak fertility. Day 11 was chosen because peak-fertility window, as shown previously, was found to occur between days 10–12. It is important to note that based on these results, thresholds around 25–30 mIU/ml had overall the best PPV (50–60%), NPV (98%), LR+ (20–30), and LR− (0.5).

**Table 3:** The sensitivity (Se), specificity (Sp), positive predictive value (PPV), confidence intervals (CI), negative predictive value (NPV), likelihood ratios +’ve (LR+) and likelihood ratios −’ve (LR−) for predicting ovulation within 24 h at 15, 20, 25, 30, 35, and 40 mIU/ml thresholds on the 11th day of the cycle.

Threshold (mIU/ml)	Sn (CI)	Sp (CI)	PPV	NPV	LR+	LR−
40	0.23 (0.08–0.50)	0.99 (0.97–1.00)	0.23	0.99	20.62	0.78
35	0.31 (0.13–0.58)	0.99 (0.97–1.00)	0.31	0.99	27.49	0.97
30	0.46 (0.23–0.71)	0.99 (0.96–0.99)	0.60	0.97	30.92	0.55
25	0.54 (0.29–0.77)	0.97 (0.95–0.99)	0.50	0.98	20.62	0.47
20	0.54 (0.29–0.77)	0.96 (0.93–0.98)	0.41	0.98	14.43	0.48
15	0.54 (0.29–0.77)	0.94 (0.91–0.98)	0.32	0.98	9.62	0.49

**Figure 5:**
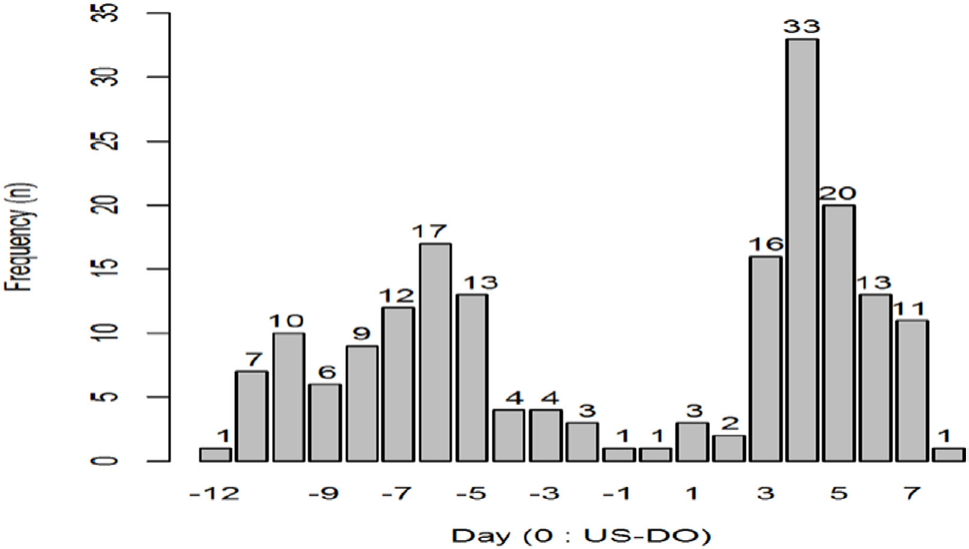
Number of cycles with one positive day [luteinizing hormone (LH) threshold ≥25 mlU/ml] followed by three consecutive negative days (LH thresholds ≤25 mlU/ml) in relation to ultrasound day of ovulation (0 US-DO).

In regard to our two tested hypothesis, Figure 5 illustrates the example of using an LH test with a threshold set at 25 mIU/ml to assess the end of the fertile window. Using our criteria, we found that 88 out of 283 cycles (31%) were found to incorrectly identify the end of the fertile window. As for our second hypothesis, as shown on Table 4, when observed concurrently and throughout the cycle, peak-type mucus with a positive LH test ≥25 mIU/ml provides the higher specificity (97–99%) and PPV (34–37%) than either mucus (77–95% and 20–29%) or LH alone (91 and 20%) to predict ovulation.

**Table 4:** The sensitivity (Se), specificity (Sp), positive predictive value (PPV), negative predictive value (NPV), and confidence intervals (CI) for predicting ovulation within 24 h by the use of Peak-fertility type mucus (Score 4), luteinizing hormone test or the combination of both from days 7 to 20 of the menstrual cycle.

Scenario	Se (CI)	Sp (CI)	PPV (CI)	NPV (CI)
First day of occurrence of Score 4 mucus	0.731 (0.675–0.781)	0.773 (0.759–0.787)	0.197 (0.173–0.223)	0.974 (0.968–0.979)
Last day of occurrence of Score 4 mucus	0.284 (0.230– 0.345)	0.948 (0.940–0.955)	0.293 (0.237–0.355)	0.946 (0.937–0.953)
First day of LH ≥ 25	0.297 (0.246–0.354)	0.912 (0.902–0.920)	0.202 (0.166–0.244)	0.945 (0.937–0.952)
First day of occurrence of Score 4 mucus and LH ≥ 25	0.223 (0.177–0.276)	0.971 (0.966–0.976)	0.370 (0.300–0.446)	0.943 (0.935–0.950)
Last day of occurrence of Score 4 mucus and LH ≥ 25	0.100 (0.070–0.142)	0.986 (0.981–0.989)	0.342 (0.247–0.452)	0.936 (0.928–0.943)

Finally, in terms of patient characteristics and lifestyles, our data did not seem to provide any major differences in terms of age, BMI, or past use of oral contraception (Figure S7 in Supplementary Material; Table 5) for predicting ovulation with the use of the LH tests. However, our population was relatively homogenous in regards to BMI: about 10% of total was <BMI 18.5 and 10% of total participants with a >BMI 25. BMI was the only variable to affect the LH levels. A higher BMI was associated with a lower LH level; yet, this did not affect its predictive value.

**Table 5:** Multivariate regression analysis of body mass index (BMI), age, past used of oral contraception, sport activity, and smoking on the natural logarithm of urinary LH levels from days 7 to 20 of the menstrual cycle.

Variable	Coefficient (95% coefficient interval)	*p*-Value
Body mass index	−0.05 (−0.09 to −0.01)	0.020
Age	0.17 (−0.01 to 0.36)	0.068
Contraception	0.20 (−0.03 to 0.43)	0.088
Sport activity	−0.20 (–0.44 to 0.04)	0.105
Currently smoking	0.16 (−0.19 to 0.51)	0.369

## Discussion

Several findings may be drawn from our study. First, testing earlier in the cycle with LH tests beginning on day 7 has better predictive value than later in the cycle. This is relevant since many manufacturer’s instructions recommend starting testing only after days 10–11. Another finding was that accuracy increased if the tests were used for a longer duration in the cycle. It is important to note that using the LH test alone to delineate the end of the fertile window may provide a false end of the fertile window and may even occur before ovulation in one out of three cycles as shown in Figure 5 (31.1% of the cycles showed positivity followed by three daily negatives even though ovulation had not yet occurred). Given the fact that LH kits are now very affordable, its daily use for several days until the end of fertile window would be recommended when trying to achieve a pregnancy. As mentioned later on, other means may be needed to ascertain the end of the fertile window.

Our study also found that, in general, once the first test becomes positive, i.e., above a given threshold, the first 24 h after this change represents the highest probability that ovulation may occur. Further positives would not necessarily imply higher probability of ovulation but may simply confirm that the peak fertile window has been reached. According to our results, thresholds around 25–30 mIU/ml may represent the best cutoff to predict ovulation during this fertile window with a PPV of 50–60% and NPV of 98%. The finding that roughly one-half to one-third of cycles with one first positive test will not predict ovulation within 24 h needs to be pointed out when advising women who are using these tests. The use of a definite confirmatory test such as urine pregnanediol test would be ideal at this point to demonstrate that ovulation has occurred (10). Based on these findings, we also recommend that manufacturers disclose their tests’ thresholds to the public.

Recently, it has been suggested that the timing of peak LH was assay-dependent and could be post-ovulatory, therefore, a simple LH test should not be the sole method used to predict/determine ovulation (16). The same authors found that “there is overlap between the population baseline value of intact LH (90th centile around 10–15 mIU/mL prior to surge) and surge level (10th centile for day of ovulation 9.9 mIU/mL),” which led to their conclusion that “a single threshold would not provide 100% accurate prediction.” We agree with these conclusions. In fact, our finding that early cycle testing increases its predictive value may be due to the ability of capturing the LH surge rather than the LH peak. The LH surge over its follicular baseline has been found to be a better marker than the LH peak to predict ovulation (16).

It would be helpful to further investigate the use of complementary markers such as cervical mucus (15, 17) or urinary PDG (10) as to identify the beginning and end of the fertile window, respectively, in individual women. We demonstrated that the combination of peak-fertility type cervical mucus (Score 4) increases the specificity and PPV of the LH test. It is important to note that despite its apparent low PPV (34–37%), its specificity is very high (97–99%). The former can be explained by two main factors. The first is that PPV is the composite result of daily testing from days 7–20 with PPV varying depending on its nearness to the ovulation day. The second is that the requirement of simultaneous occurrence of both LH ≥25 and mucus score 4 lowers the PPV (34–37%) as well as its Sensitivity (10–22%). On the other hand, if both markers are experienced, its specificity for ovulation prediction is excellent (97–99%). We view PPV and Se as both playing an important role for women trying to predict ovulation. Differently from the use of diagnosis tests for illness detection, seeking a higher sensitivity is important in order to increase the proportion of ovulations being identified by the LH test. PPV is also important because a higher proportion of predicted ovulations will indeed take place the day after the positive test. As a result, we would recommend as possible future research to test a hypothesis whether Se and PPV may increase depending on the order of occurrence of the markers, such as Mucus score 4 first then a LH positive.

Other commonly used markers such as basal body temperature may be useful as well for the end of the fertile window (18) but may be less precise (19). In addition, development of urinary hormone monitors that provide quantitative results for various hormones may give relevant detailed information to determine whether an individual woman’s levels were changing from her particular baseline (19, 20).

In our dataset, we found that higher BMI was significantly correlated with lower LH levels. Women with higher BMI may have lower LH levels, and hypothetically, a positive test may be less frequent. This finding has the possibility to decrease the sensitivity and improve specificity depending on the threshold. Table 5 shows that other potential confounders might exist such as age on past use of contraception given that the *p*-values were not far from statistically significance of 0.05. This opens to a variability of Se and Sp and, thus, to PPV and NPV among women. Further research may be advised.

As highlighted by our study and others (8, 21), LH tests have some confounding factors affecting how they predict ovulation such as the timing of the test during the cycle, the quality of the lateral flow assay itself, the ease of interpretation by the user, the threshold of the test, and some biological conditions such as Luteinized Unruptured Follicles (LUFs) and the variability of LH secretion in individual women.

The main strength of our study was the large number of cycles with ultrasound-confirmed ovulation that could be correlated with daily urinary assays. The main limitation of the study relates to the generalizability since we excluded anovulatory cycles and potential LUFs, in addition to the homogeneity of the study population as demonstrated by BMI and demographics.

## Conclusion

In our study, we identified that beginning LH testing earlier in the cycle (day 7) with a threshold of 25–30 mIU/ml may present the best predictive value for ovulation within 24 h. However, prediction by LH testing alone may be affected negatively by several confounding factors so LH testing alone should not be used to define the end of the fertile window. Complementary markers should be further investigated to predict ovulation and identify the fertile window. The use of the peak cervical mucus along with an LH test may provide a higher specificity and predictive value than either of them alone. We recommend that manufacturers disclose their tests’ threshold to the public.

## Ethics Statement

The study was approved by the local Ethics Committee (Comite Consultatif de Protection des Personnes dans la Recherche Biomedicale de Lyon). Each of the participants gave their written informed consent, and the study procedures were carried out in accordance with the Ethical Standards for Human Experimentation established by the Declaration of Helsinki.

## Author Contributions

RL conceived the study. RL, TB, and RE designed the analysis, analyzed the data, and wrote the manuscript. RE and SA performed the statistical analysis.

## Conflict of Interest Statement

The authors declare that the research was conducted in the absence of any commercial or financial relationships that could be construed as a potential conflict of interest.
