# Corrigendum: Urinary Luteinizing Hormone Tests: Which Concentration Threshold Best Predicts Ovulation?

**DOI:** 10.3389/fpubh.2018.00345

**Published:** 2018-11-30

**Authors:** Rene Antonio Leiva, Thomas Paul Bouchard, Saman Hasan Abdullah, René Ecochard

**Affiliations:** ^1^Bruyère Research Institute and C. T. Lamont Primary Health Care Research Centre, Department of Family Medicine, University of Ottawa, Ottawa, ON, Canada; ^2^Department of Family Medicine, University of Calgary, Calgary, AB, Canada; ^3^Service de Biostatistique, Hospices Civils de Lyon, Lyon, France; ^4^Université de Lyon, Lyon, France; ^5^Université Lyon 1, Lyon, France; ^6^Équipe Biostatistique-Santé, Laboratoire de Biométrie et Biologie Évolutive, Centre National de la Recherche Scientifique, Unité Mixte de Recherche 5558, Villeurbanne, France

**Keywords:** ovulation predictor kits, luteinizing hormone, natural family planning, fertility awareness methods, infertility, urine, ovulation, fertile window

In the original article, there was a mistake in Table 3 “The sensitivity (Se), specificity (Sp), positive predictive value (PPV), confidence intervals (CI), negative predictive value (NPV), likehood ratios +'ve (LR+) and likehood ratios –'ve (LR-) for predicting ovulation within 24 h at 15, 20, 25, 30, 35, and 40 mIU/ml thresholds on the 11th day of the cycle”. Upon review of the tables prior to a journal club, it was noted that there were some numerical values that had been inadvertently misplaced under the wrong columns when updating different previously edited tables. The corrected Table 3 appears below.

The authors apologize for this error and state that this does not change the scientific conclusions of the article in any way. The original article has been updated.

**Table 3:** The sensitivity (Se), specificity (Sp), positive predictive value (PPV), confidence intervals (CI), negative predictive value (NPV), likehood ratios +'ve (LR+) and likehood ratios −'ve (LR−) for predicting ovulation within 24 h at 15, 20, 25, 30, 35, and 40 mIU/ml thresholds on the 11th day of the cycle.

**Threshold (mIU/ml)**	**Sn (CI)**	**Sp (CI)**	**PPV**	**NPV**	**LR+**	**LR–**
40	0.23 (0.08–0.50)	0.99 (0.97–1.00)	0.23	0.99	20.62	0.78
35	0.31 (0.13–0.58)	0.99 (0.97–1.00)	0.31	0.99	27.49	0.97
30	0.46 (0.23–0.71)	0.99 (0.96–0.99)	0.60	0.97	30.92	0.55
25	0.54 (0.29–0.77)	0.97 (0.95–0.99)	0.50	0.98	20.62	0.47
20	0.54 (0.29–0.77)	0.96 (0.93–0.98)	0.41	0.98	14.43	0.48
15	0.54 (0.29–0.77)	0.94 (0.91–0.98)	0.32	0.98	9.62	0.49

## Conflict of interest statement

The authors declare that the research was conducted in the absence of any commercial or financial relationships that could be construed as a potential conflict of interest.

