# Supplementary material for: Urinary Luteinizing Hormone Tests: Which Concentration Threshold Best Predicts Ovulation?
Source: Front Public Health. 2017 Nov 28;5:320. doi: 10.3389/fpubh.2017.00320 (PMC5712333; doi:10.3389/fpubh.2017.00320)
Supplement: Supplementary file 2 [file Presentation_1.pdf]

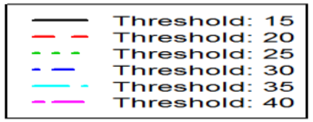

Supplemental Figure 6. The Sensitivity (Se), Specificity (Sp), Positive Predictive value (PPV), Negative Predictive value (NNV) ,Likehood Ratios +'ve (LR+) and Likehood Ratios -'ve (LR-) for the 15, 20, 25, 30, 35 and 40 mIU/ml thresholds across the menstrual cycle

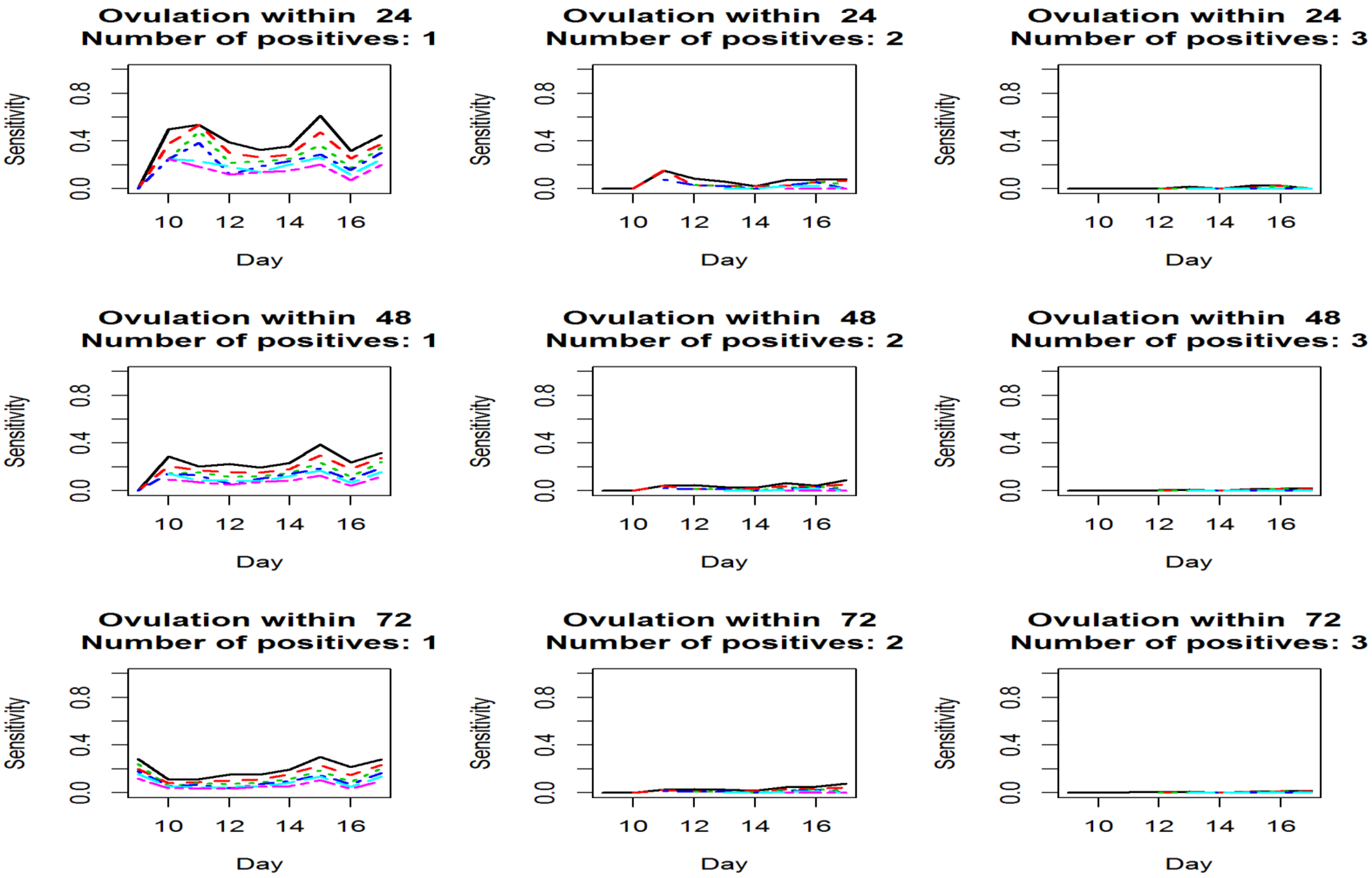

Ovulation within 24
Number of positives: 1
Ovulation within 24
Number of positives: 2
Ovulation within 24
Number of positives: 3
Ovulation within 48
Number of positives: 1
Ovulation within 48
Number of positives: 2
Ovulation within 48
Number of positives: 3
Ovulation within 72
Number of positives: 1
Ovulation within 72
Number of positives: 2
Ovulation within 72
Number of positives: 3
Sensitivity
0.0
0.4
0.8
Day
10
12
14
16

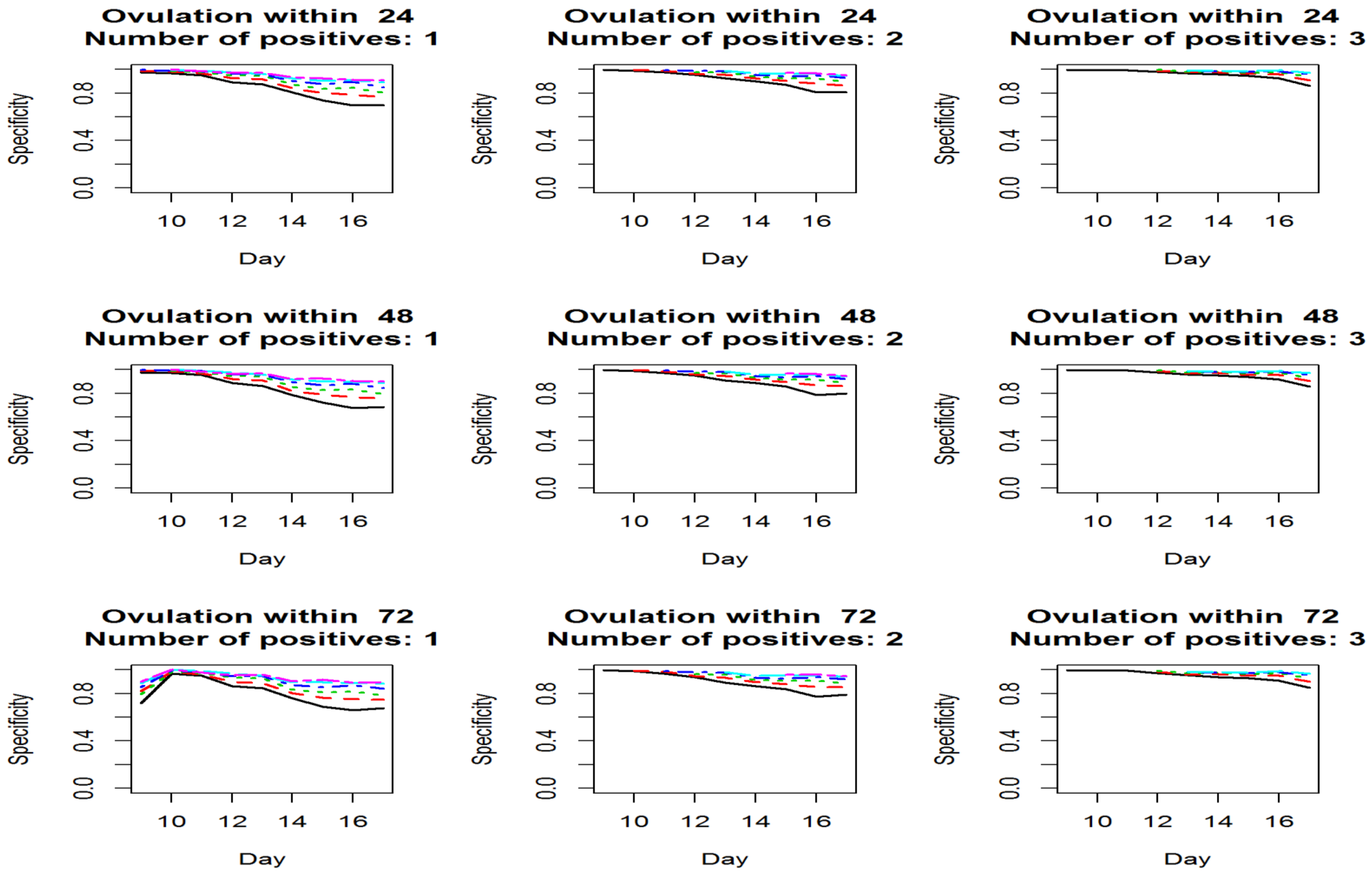
Ovulation within 24
Number of positives: 1
Ovulation within 24
Number of positives: 2
Ovulation within 24
Number of positives: 3
Ovulation within 48
Number of positives: 1
Ovulation within 48
Number of positives: 2
Ovulation within 48
Number of positives: 3
Ovulation within 72
Number of positives: 1
Ovulation within 72
Number of positives: 2
Ovulation within 72
Number of positives: 3
Specificity
0.0
0.4
0.8
10
12
14
16
Day

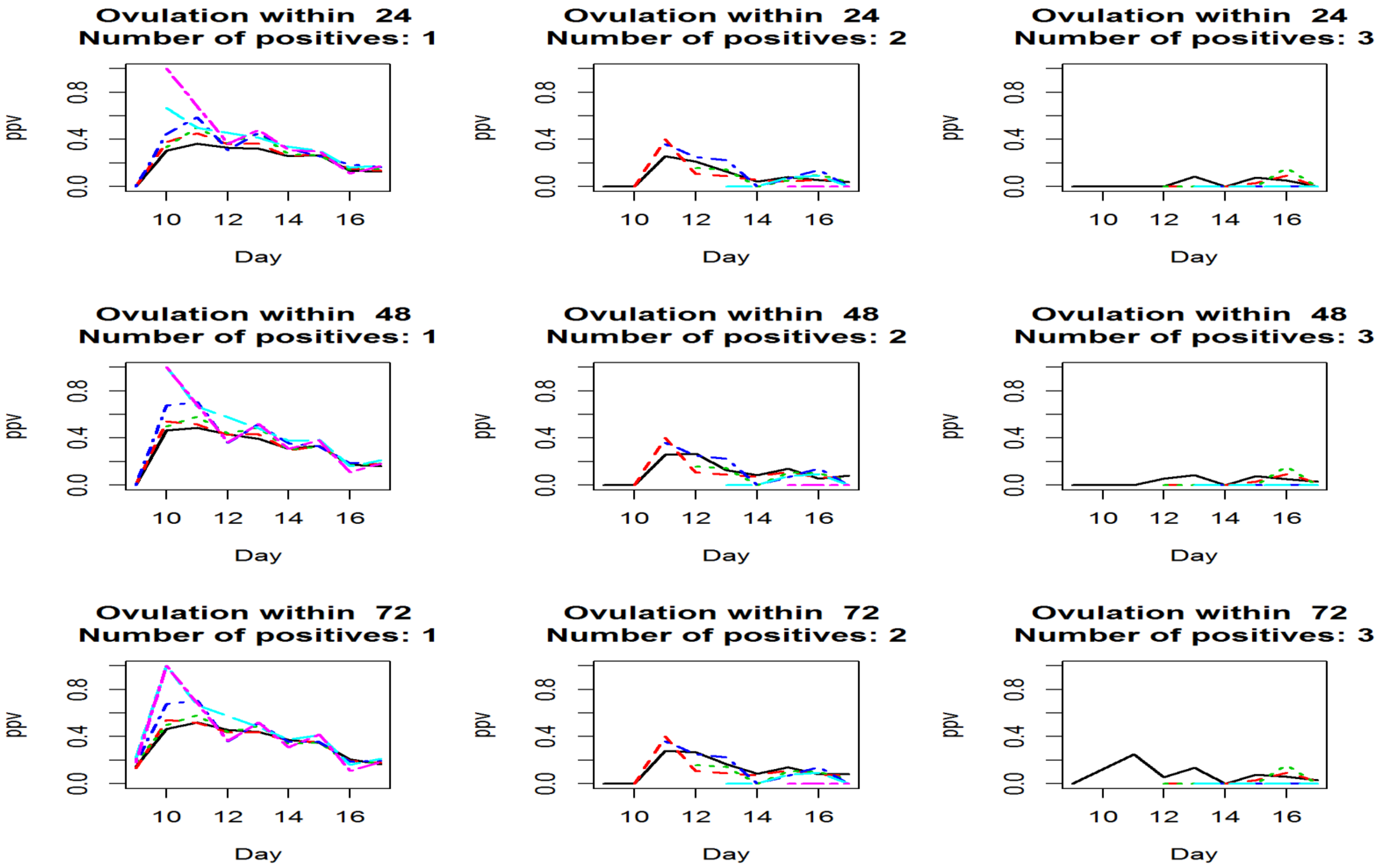
Ovulation within 24
Number of positives: 1
Ovulation within 24
Number of positives: 2
Ovulation within 24
Number of positives: 3
Ovulation within 48
Number of positives: 1
Ovulation within 48
Number of positives: 2
Ovulation within 48
Number of positives: 3
Ovulation within 72
Number of positives: 1
Ovulation within 72
Number of positives: 2
Ovulation within 72
Number of positives: 3
ppv
0.0
0.4
0.8
Day
10
12
14
16

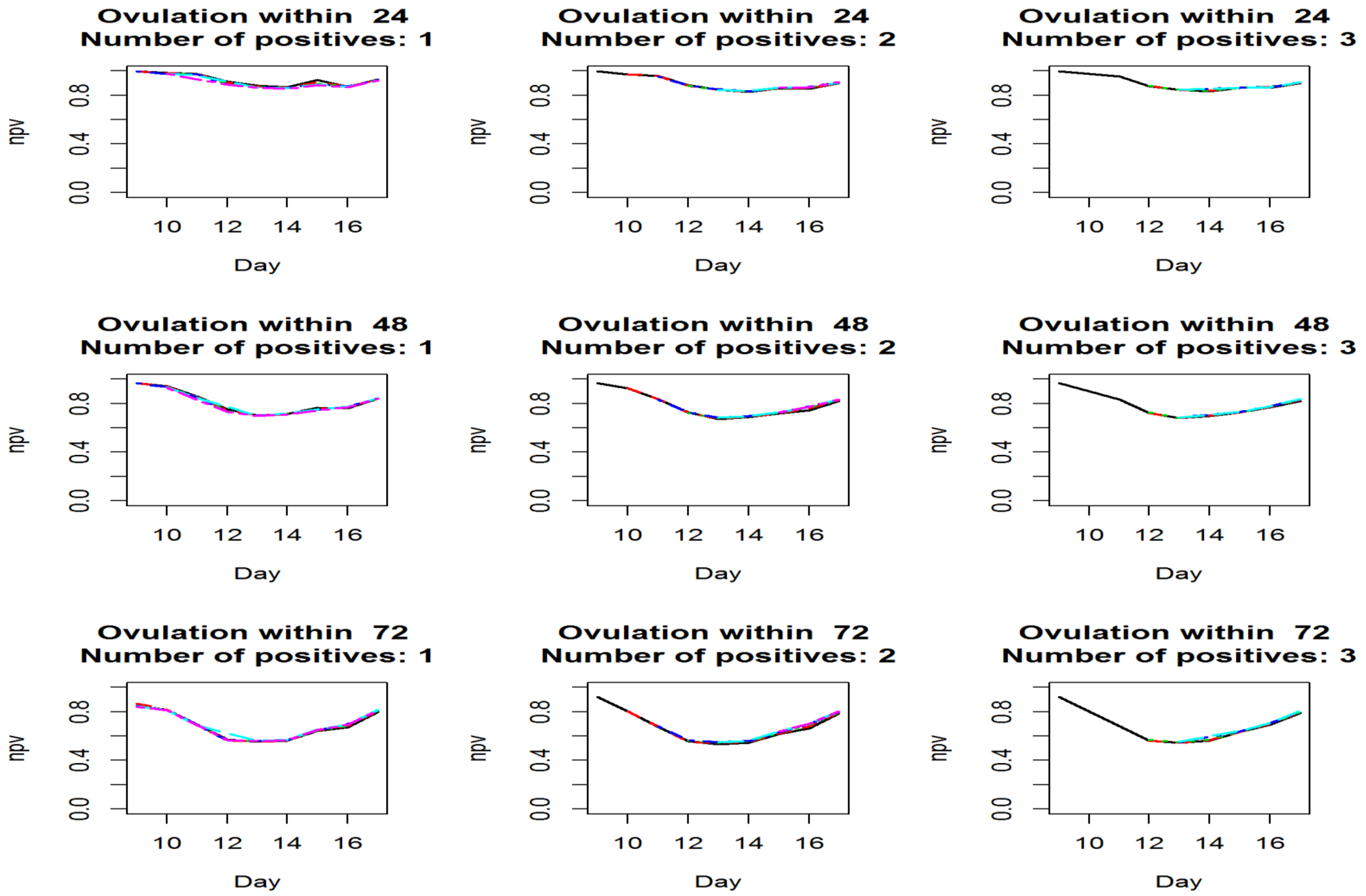
Ovulation within 24
Number of positives: 1
Ovulation within 24
Number of positives: 2
Ovulation within 24
Number of positives: 3
Ovulation within 48
Number of positives: 1
Ovulation within 48
Number of positives: 2
Ovulation within 48
Number of positives: 3
Ovulation within 72
Number of positives: 1
Ovulation within 72
Number of positives: 2
Ovulation within 72
Number of positives: 3
npv
0.0
0.4
0.8
Day
10
12
14
16

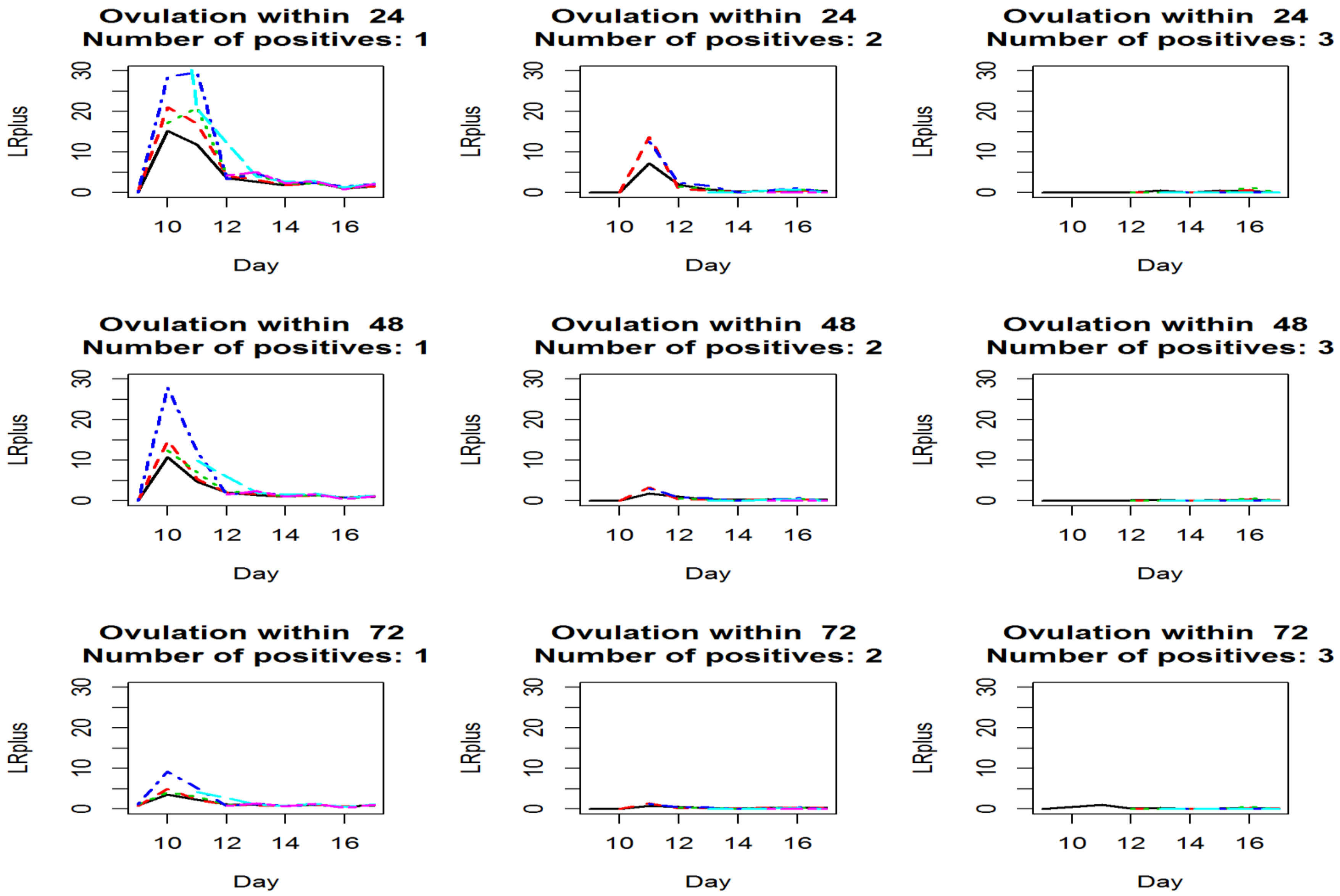
Ovulation within 24
Number of positives: 1
Ovulation within 24
Number of positives: 2
Ovulation within 24
Number of positives: 3
Ovulation within 48
Number of positives: 1
Ovulation within 48
Number of positives: 2
Ovulation within 48
Number of positives: 3
Ovulation within 72
Number of positives: 1
Ovulation within 72
Number of positives: 2
Ovulation within 72
Number of positives: 3
LRplus
Day
0
10
20
30
10
12
14
16

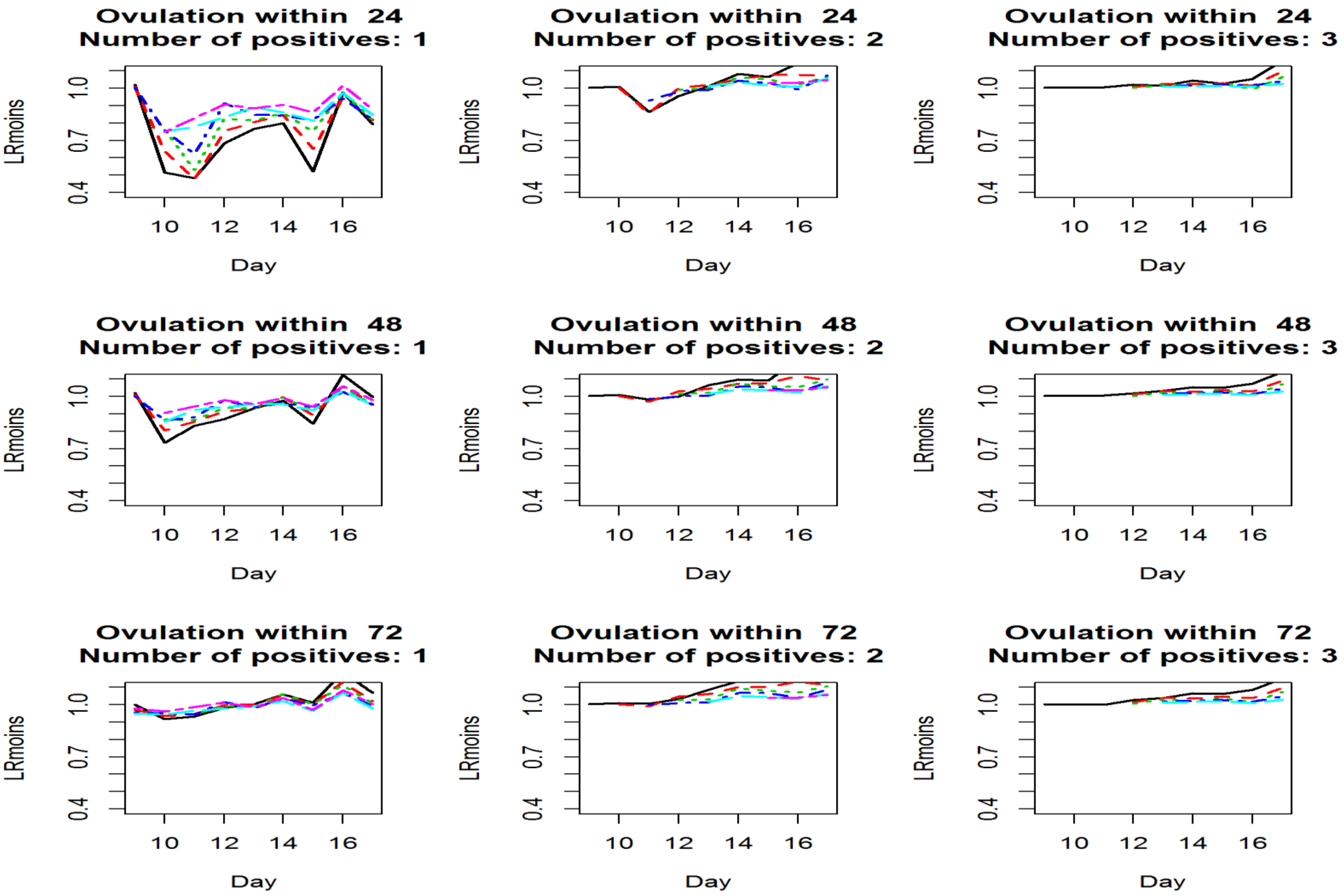
Ovulation within 24
Number of positives: 1
Ovulation within 24
Number of positives: 2
Ovulation within 24
Number of positives: 3
Ovulation within 48
Number of positives: 1
Ovulation within 48
Number of positives: 2
Ovulation within 48
Number of positives: 3
Ovulation within 72
Number of positives: 1
Ovulation within 72
Number of positives: 2
Ovulation within 72
Number of positives: 3
LRmoins
Day
0.4
0.7
1.0
10
12
14
16
