# Supplementary material for: Urinary Luteinizing Hormone Tests: Which Concentration Threshold Best Predicts Ovulation?
Source: Front Public Health. 2017 Nov 28;5:320. doi: 10.3389/fpubh.2017.00320 (PMC5712333; doi:10.3389/fpubh.2017.00320)
Supplement: Supplementary file 3 [file Presentation_2.pdf]

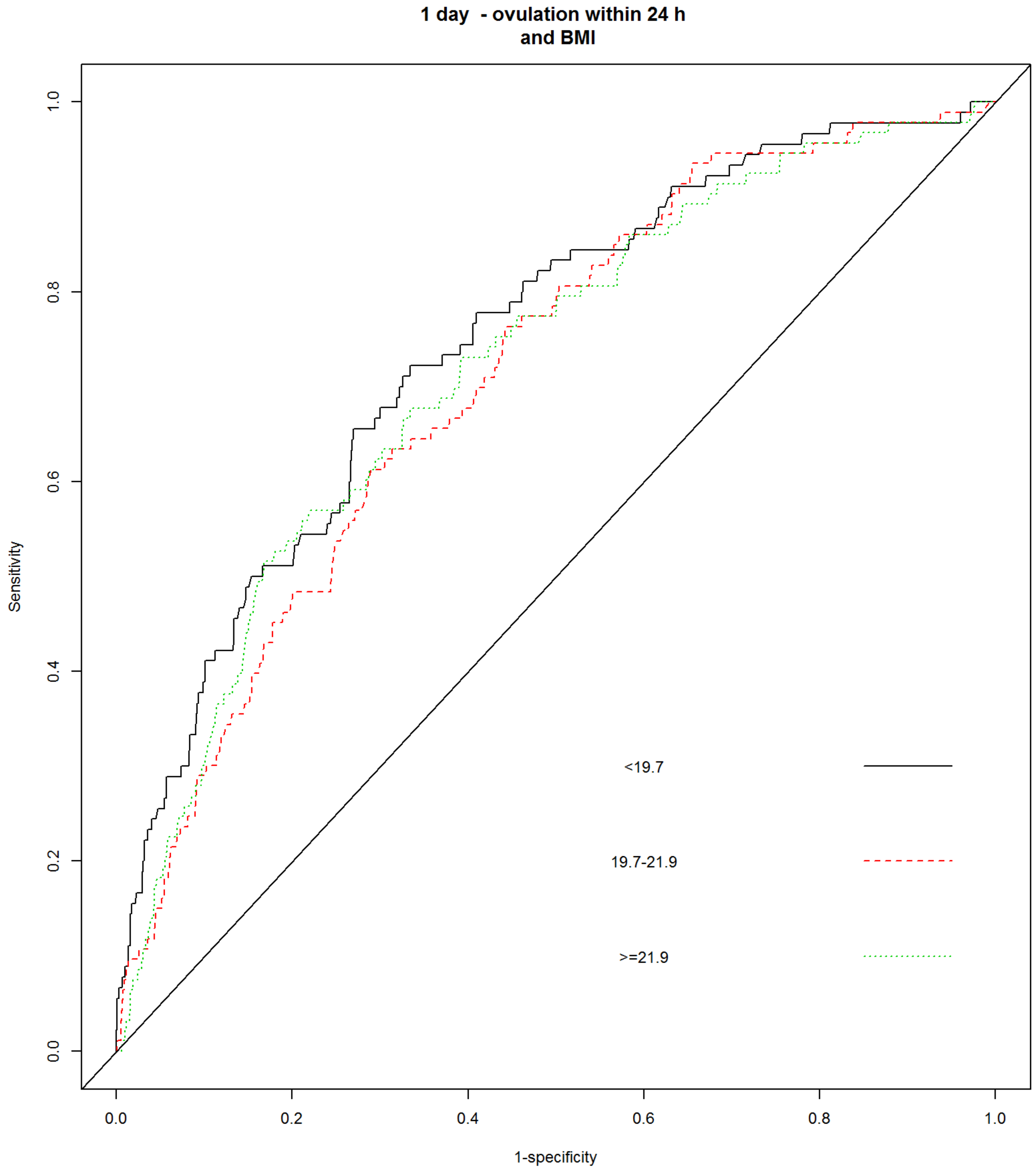

Figure A. The Receiver Operating Characteristic curves for one random positive LH test to predict ovulation within 24 hours- across the menstrual cycle- applying different ranges on BMI (<19.7; 19.7-21.9; ≥21.9).

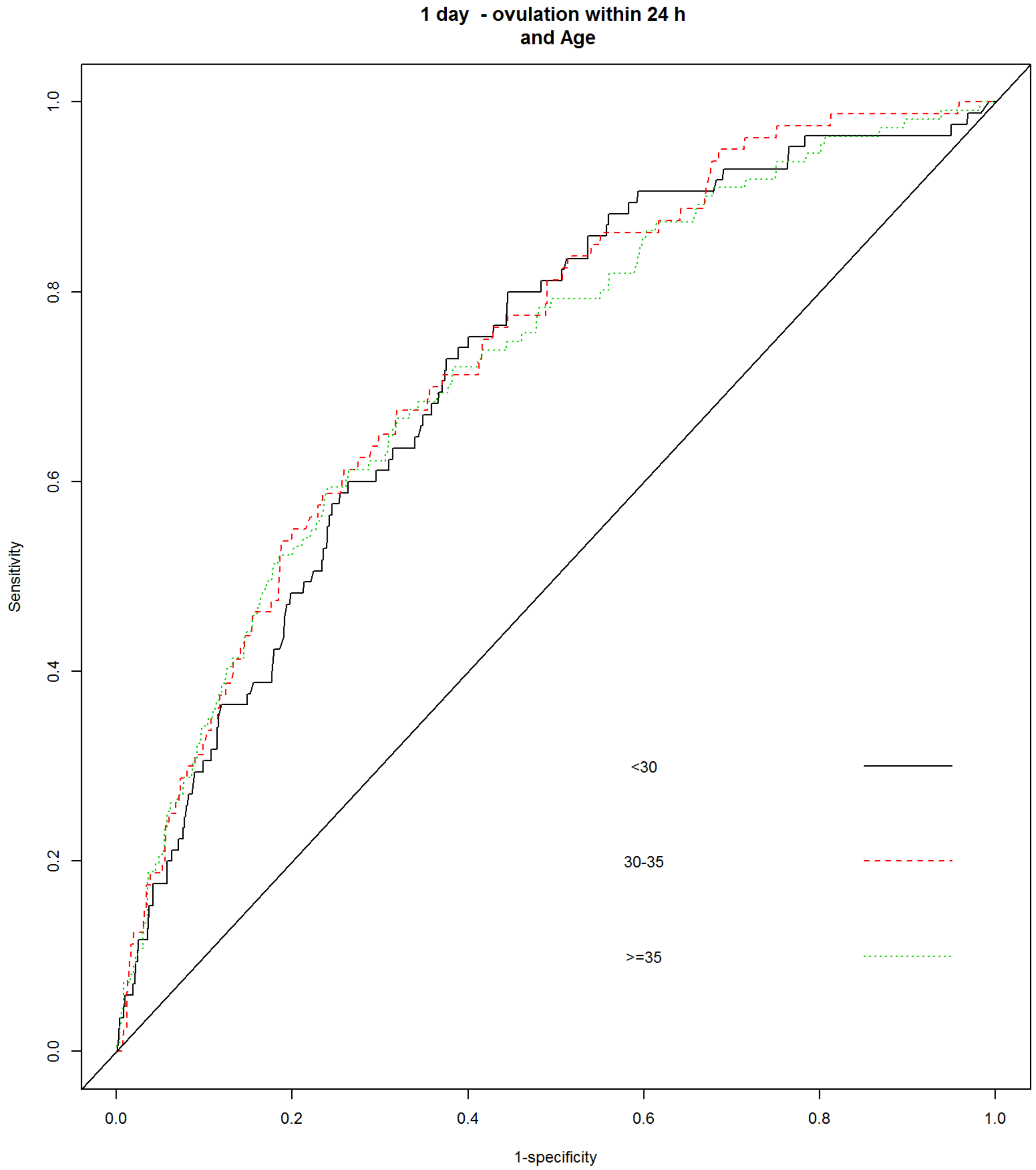

Figure B. The Receiver Operating Characteristic curves for one random positive LH test to predict ovulation within 24 hours- across the menstrual cycle- applying different ranges based on age-years (<30; 30-35; ≥35).

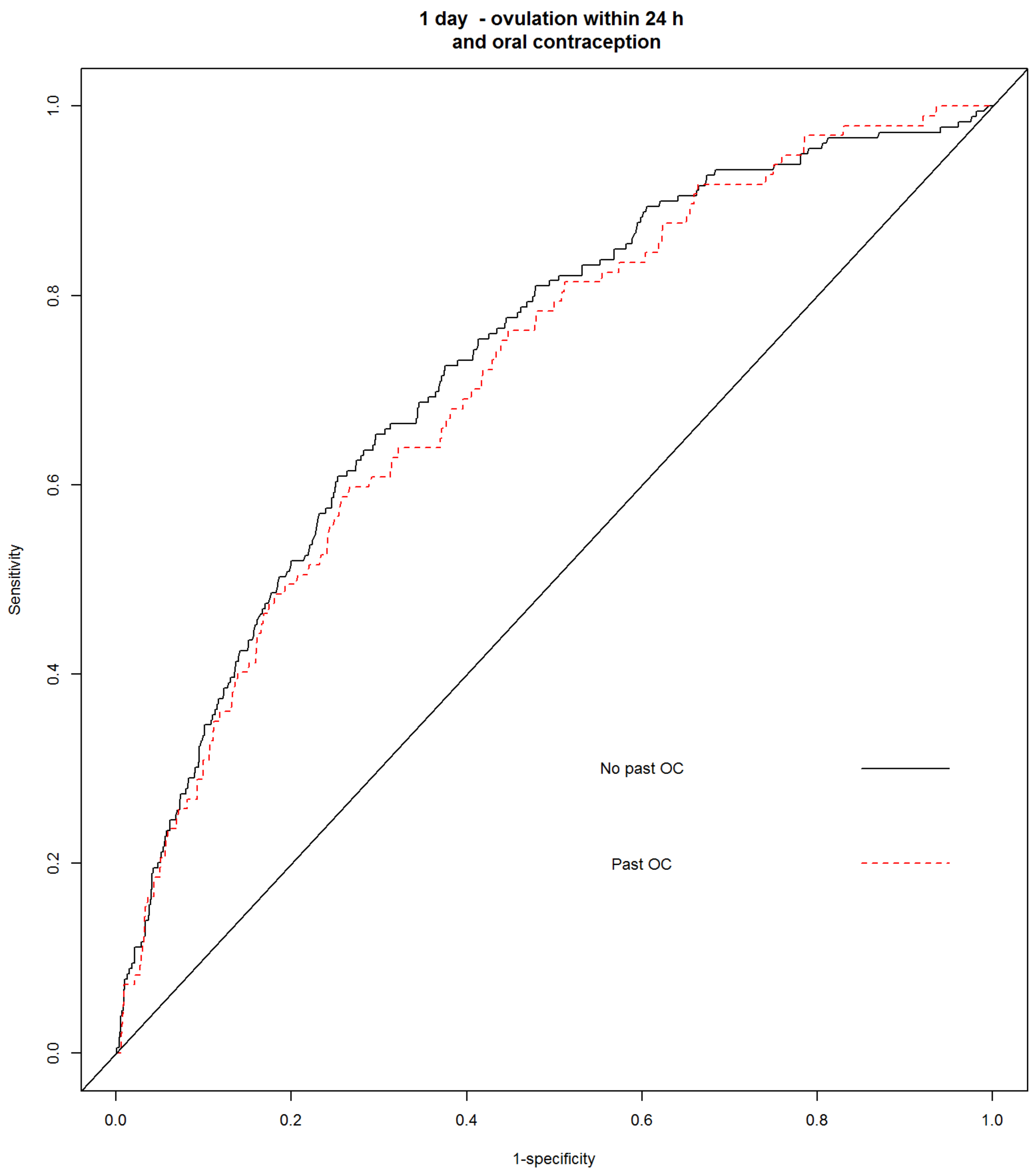

Figure C. The Receiver Operating Characteristic curves for one random positive LH test to predict ovulation within 24 hours- across the menstrual cycle- applying past use of oral contraception.
